# Imaginal disc growth factor 4 regulates development and temperature adaptation in *Bactrocera dorsalis*

**DOI:** 10.1038/s41598-018-37414-9

**Published:** 2019-01-30

**Authors:** Xinyue Gu, Zhihong Li, Yun Su, Yan Zhao, Lijun Liu

**Affiliations:** Department of Entomology, College of Plant Protection, China Agricultural University, Beijing, 100193 China

## Abstract

The oriental fruit fly *Bactrocera dorsalis* (Hendel) (Diptera: Tephritidae) is an important invasive pest with high reproductive capacity and invasiveness; it has shown remarkable range expansion and brings higher risk to the environment and agriculture. The insect cuticle serves as skin and skeleton, protecting insects against numerous harmful stresses. One gene named imaginal disc growth factor 4 (*idgf4*) which is involved in cuticle formation, plays an important role in organizing proteins in the chitin-matrix, as well as in adult molting. This gene in the poorly-described glycoside hydrolase 18 (GH 18) family was chosen to study the function of chitinases in insect defense barrier against heat and molting using quantitative real-time PCR (qRT-PCR) and RNA interference (RNAi). qRT- PCR showed that *idgf4* was expressed in all nine developmental stages and was mainly expressed in the early and late pupal, as well as adult stages. Knocking down the *idgf4* gene via RNAi in 3^rd^ instar larvae led to the decreased survival of larvae under high temperatures and malformed individuals as adults. The results indicated the function of the *idgf4* gene in the fruit fly’s defense barrier and development. It can provide new insights into understanding the function of one member in the GH 18 family, and may reveal a new potential gene for pest control.

## Introduction

Tephritid flies attack a large variety of fruits, which can be highly priced commodities in many countries^1^. The oriental fruit fly *Bactrocera dorsalis* is an important invasive Tephritid pest that causes major financial losses in the fruit and horticultural industries because of its high reproductive capacity and invasiveness^2–4^. Over the past 10 years, *B. dorsalis* has shown remarkable range expansion and invaded several new continents^5^. As noted in recent studies, four major pest species, *B. dorsalis*, *B. philippinensis*, *B. papayae* and *B. invadens*, belong to a *B. dorsalis* complex, which makes it more widely distributed and a higher risk than previously thought^6^. Especially against the background of climate change, the potential distribution of *B. dorsalis* increases significantly in the projected scenarios^7^. Considering its role in high levels of economic loss and an expanding wider distribution, several methods, including destruction of the fallen and infested fruits, cold and heat treatments, bait sprays, biological control methods, sterile insect techniques (SIT) and usage of insecticides have been developed in the last few decades for controlling this notorious pest^8–15^. Among all these methods, insecticides still represent the most effective strategy^15,16^. However, due to the rapid development of insecticide resistance, the control of oriental fruit flies has become difficult^7^. The usefulness of RNA interference (RNAi) in functional genomic research in insects and its considerable potential for the control of pest insects has recently been suggested^17^. Moreover, RNAi can also be used effectively in some classic methods of insect control, such as SIT^18,19^. Using an RNAi approach successfully for pest control mainly relies on effective target gene selection, and it is of great significance to find the potential target genes for the development of this new technology in *B. dorsalis*^20^.

The glycoside hydrolase 18 (GH 18) family of chitinases is a key family in insects that plays various roles in insect cuticle development and molting^21–23^. Based on phylogenetic analyses, the GH 18 family is encoded by a large number of diverse genes and can be classified into five groups^24^. Imaginal disc growth factor (IDGFs), belonging to the group V chitinase, were first identified by fractionating conditioned medium from *Drosophila* imaginal disc cell cultures^25,26^. Imaginal disc growth factor (IDGFs) were confirmed to be the proteins cooperating with insulin that promote the growth of cell lineages derived from imaginal discs in *D. melanogaster*^25,27,28^. Additionally, this type of cooperation with insulin can be found in other species, such as *Mamestra brassicae*^29^. In a recent study, the five nonenzymatic idgfs (*idgf1*,*3*,*4*,*5*,*6*) in *Drosophila* were certified as structural protein genes to maintain the epithelial apical extracellular matrix (ECM) scaffold against chitinolytic degradation, and also participate in many vital physiological processes of insects such as adult eclosion, development regulation and blood sugar reduction^22,30–34^.

Several studies have focused on the function of individual *idgf* genes or IDGF proteins in different species^22, 25,31,32,35–38^. In the functional study of *idgf* genes, after the individual *idgf1*, *idgf3*, *idgf4*, *idgf5* and *idgf6* genes were knocked down via RNAi in *Drosophila*, the larvae displayed serious epidermal lesions and narrowed ECM thickness, as well as a deformed epidermal chitin-matrix of varying degrees^22^. In addition, starvation was found to cause a decline in the expression level of one *idgf* gene in the blood of larvae in silkworms, which suggests that this gene may reduce the blood sugar, and thus can be used for screening human hypoglycemic drugs^32^. In the functional study, one purified IDGF protein was demonstrated an extremely high level throughout the rapid growth period of organs in *Bombyx mori*^35^. *In vitro* cell growth tests showed that, in combination with insulin, recombinant IDGF1 or IDGF2 proteins greatly stimulated the growth of cultured imaginal disk cells in *Drosophila*^25,38^. Interestingly, there are several factors like the diet and nutrition, which can regulate the secretion of one IDGF in silkworms. A high-glucose diet can suppress insect growth, however, IDGF has been confirmed to be involved in regulating the development of the midgut under this unusual diet condition^37^. Although a large number of studies have generally focused on the function in larval stages, only two related papers were founded about another important developmental stage: pupae. In *Tribolium castaneum*, ds*idgf4* injected into penultimate or last instar larvae resulted in normal pupation but caused death during adult eclosion^31^. In *B. mori*, proteins with a critically different expression profile between wild type and scaleless wing mutants were verified and revealed that one *idgf* gene was related to the development and differentiation of scale cells^36^.

The efficient development of the cuticle which is a complex exoskeleton with diverse functions, leads to the evolutionary success of insects. The insect cuticle that formed from structural cuticle proteins and chitin serves both as skin and skeleton, protecting insects against invading pathogens and numerous other harmful stresses^22,39^. Several studies have shown that healthy and dark cuticles are associated with invasive abilities such as temperature adaptation and desiccation resistance^40–44^. In a major destructive insect pest called *Ceratitis capitata*, in-depth curation of more than 1800 mRNAs showed that specific gene expansions could be related to invasiveness and host adaptation, and gene families of cuticle proteins are important families among these genes^40^. Dark color cuticles in *Drosophila* are associated with reduced water loss rates^41,42^. *D. suzukii* ‘winter morph’ adults with darkened cuticles captured in temperate regions in autumn are the most cold-tolerant form^44^. Meanwhile, the cold tolerance and low temperature activity of winter-acclimated flies is related to increased cuticle melanization^43^.

In summary, previous studies of *idgf* genes have paid considerable attention to their function in larval development stages. However, there are few related researches about gene function on stress tolerance or the effect of this gene on pupal development stages. Moreover, studies often focus on the functional study of model species such as *Drosophila* species and *B. mori*^22,25,32,35–37^. There are few studies on the GH 18 family in nonmodel species, such as Tephritidae. Besides, from our previous transcriptome analysis (available RNA-seq data at NCBI: SRP093863), we found only *idgf4* and *idgf6* in the GH 18 family were differentially expressed between two *Bactrocera* species, *B. dorsalis* and *B. correcta*, in the most sensitive development stage of 3^rd^ instar larvae. *B. dorsalis* showed stronger heat plasticity than *B. correcta*, which *idgf4* may play some roles in the adaptation^45^. In summary, this study focused on the *idgf4* gene, of which little was known especially in nonmodel organisms such as *B. dorsalis*. The expression pattern with qRT-PCR and gene function of *idgf4* with RNAi on heat tolerance in the larval stage and development in the pupal stage was investigated for the first time in Tephritid fruit fly species, and provided some information on functional study and pest control in other *Bactrocera* species.

## Results

### Cloning and characterization of *idgf4*

**Figure 1:**
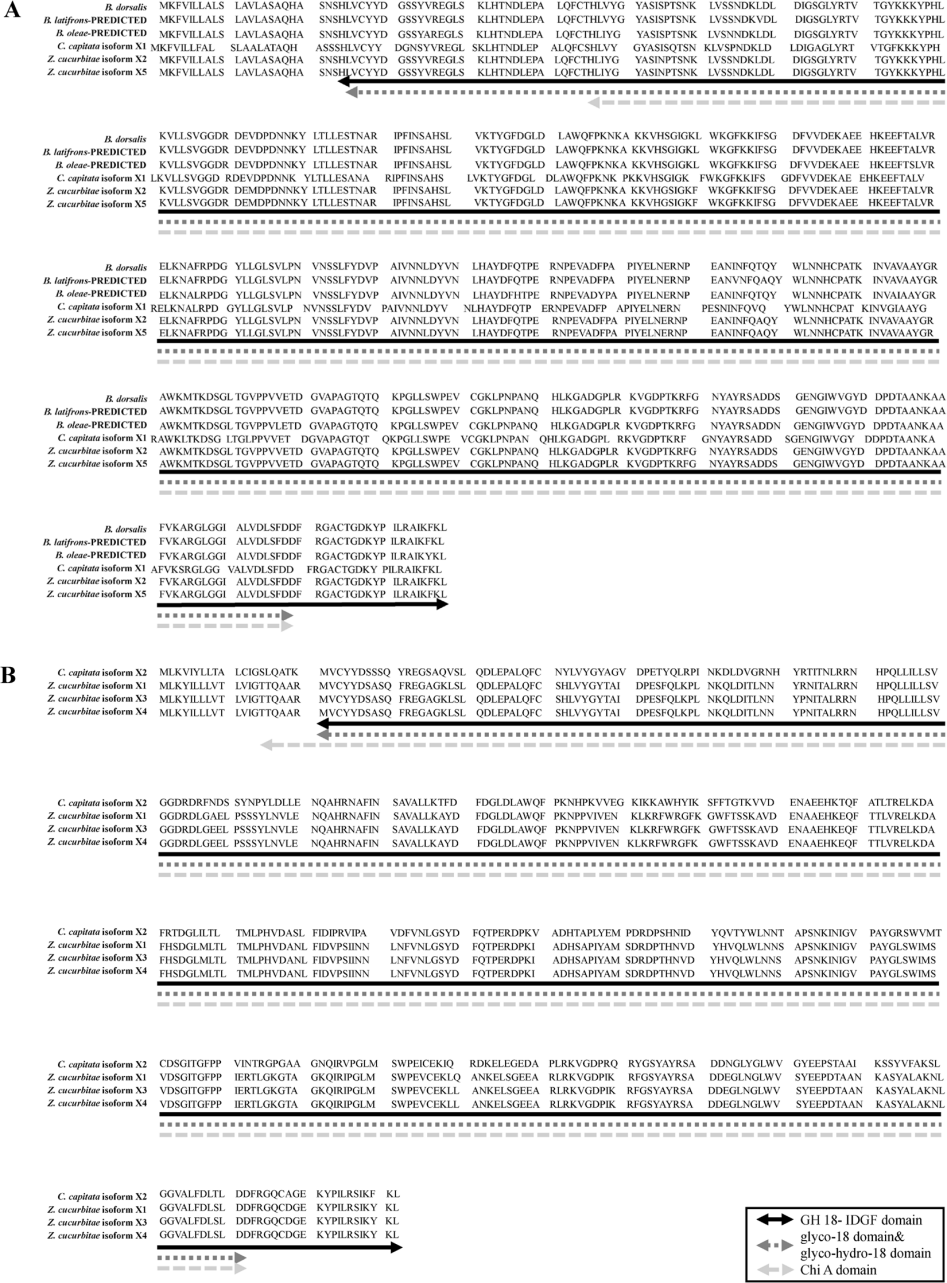
Protein sequence alignments of all the *idgf4* proteins in the Tephritid fruit flies based on NCBI BLAST results. (**A**) The protein sequence alignments using sequences similar to that of *B. dorsalis*. (**B**) The protein sequence alignments using sequences different from that of *B. dorsalis*. The alignments that present three predicted and conserved domains contained one GH 18- IDGF domain, one glyco-18 domain (glyco-hydro-18 domain) and one Chi A domain.

**Figure 2:**
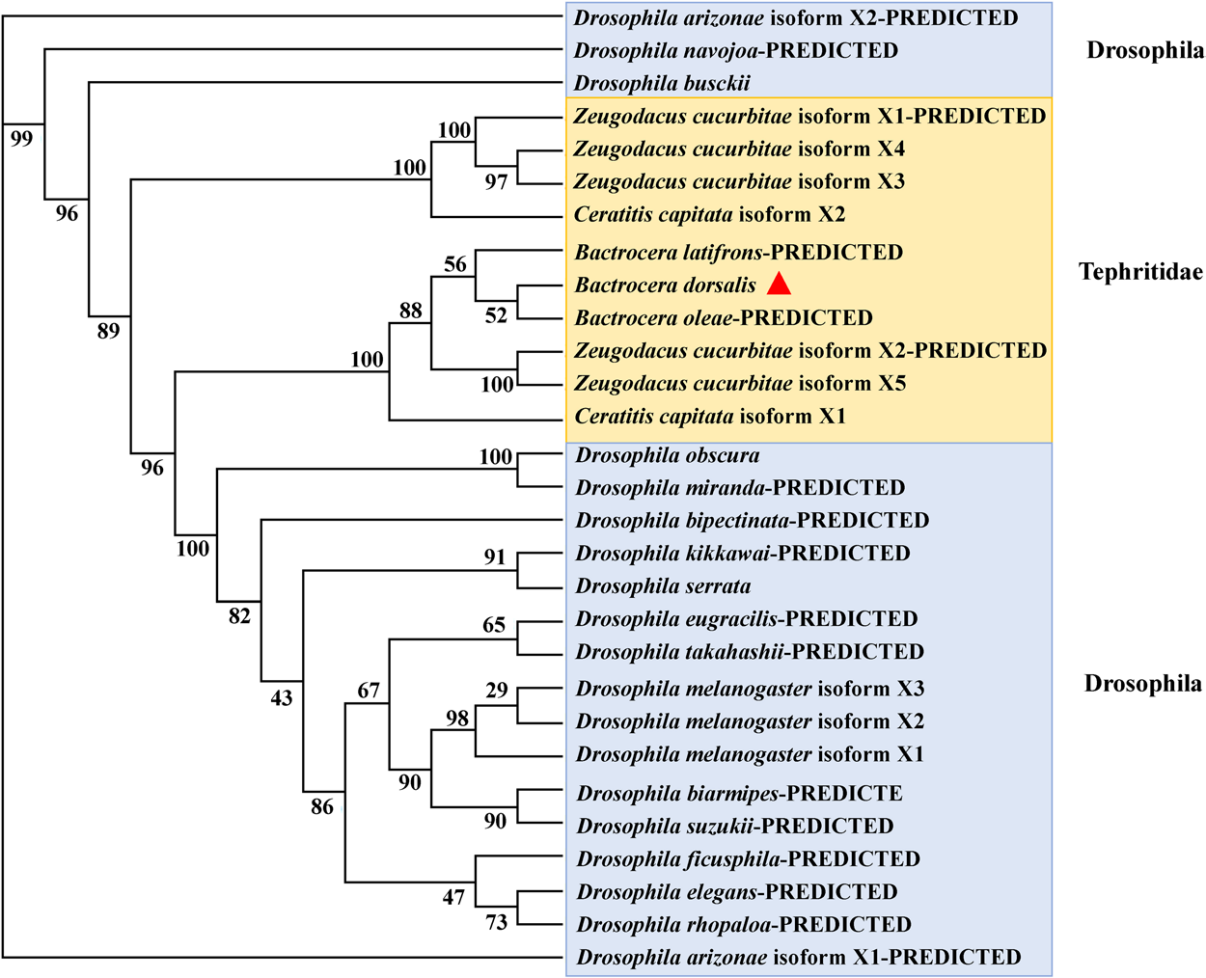
The phylogenetic analysis of *idgf4* using maximum-likelihood method in RAxML. ModelGenerator 0.85 was used to find the optimal amino acid substitution model and the LG + G model was selected. One thousand Bootstrap iterations were conducted to obtain branch support values. The *B. dorsalis idgf4* we got is labeled with a red triangle. The amino acid and nucleotide sequences were downloaded from NCBI. The accession numbers of the genes are designated with the corresponding abbreviations and are listed in Table S1.

*Idgf4* (GenBank accession no. MH 250169) was cloned from *B. dorsalis*. The open reading frame (ORF) was 1320 bp encoding 439 amino acids with a calculated molecular mass of 48.369 kDa and a theoretical isoelectric point (pI) of 7.16. To analyze the phenogenetic relationships of *idgf4* between *B. dorsalis* and other species, *idgf4* gene sequences from 21 species in *Drosophila* and Tephritidae (Table S1) were collected by BlastP in GenBank. The conserved domains of *idgf4* protein were predicted using NCBI BLAST and the protein contained one GH 18-IDGF domain, one glyco-18 domain (glyco-hydro-18 domain) and one Chi A domain (Fig. 1). Among all the Tephritid fruit flies, we found that there were two types of the genes with three same domains as same as the phylogenetic tree (Figs 1 and 2). Nucleotide sequence analysis revealed that the *idgf4* of *B. dorsalis* had the highest identity with a homolog from *B. latifrons* (96%), followed by those from *B. oleae* (93%), *Zeugodacus cucurbitae* (90%), and *C. capitate* (90%). Compared to the similar *Drosophila* species, the sequence had a highest identity of *idgf4* with *D. kikkawai* (79%). To investigate the evolutionary relationship between *idgf4* in *B. dorsalis* and other *idgf4*s, a phylogenetic analysis was performed with two different methods, the neighbor-joining and the maximum-likelihood methods. The phylogenetic tree indicated the relationship between insect *idgf4*s (Figs 2 and S1). It showed that all *idgf4*s were highly conserved among the similar species. The two methods showed the similar results, in which the *idgf4* in *B. dorsalis* was clustered closed to the other two *Bactrocera* species (Figs 2 and S1). Interestingly, two species in Tephritidae, five isoforms of *idgf4* in *Z. cucurbitae* as well as two in *C. capitata* formed two groups. One group in *Z. cucurbitae* was similar to *C. capitata* isoform X2, and one was more similar to the *Bactrocera* group (Figs 2 and S1). Apart from three species in *Drosophila*, the other species were clustered together and divided into two big groups. The amino acid sequence alignment and evolutionary relationship suggested that *idgf4*s were highly conserved among other *Bactrocera* species.

### Expression of *idgf4* in nine different development stages of *B. dorsalis*

**Figure 3:**
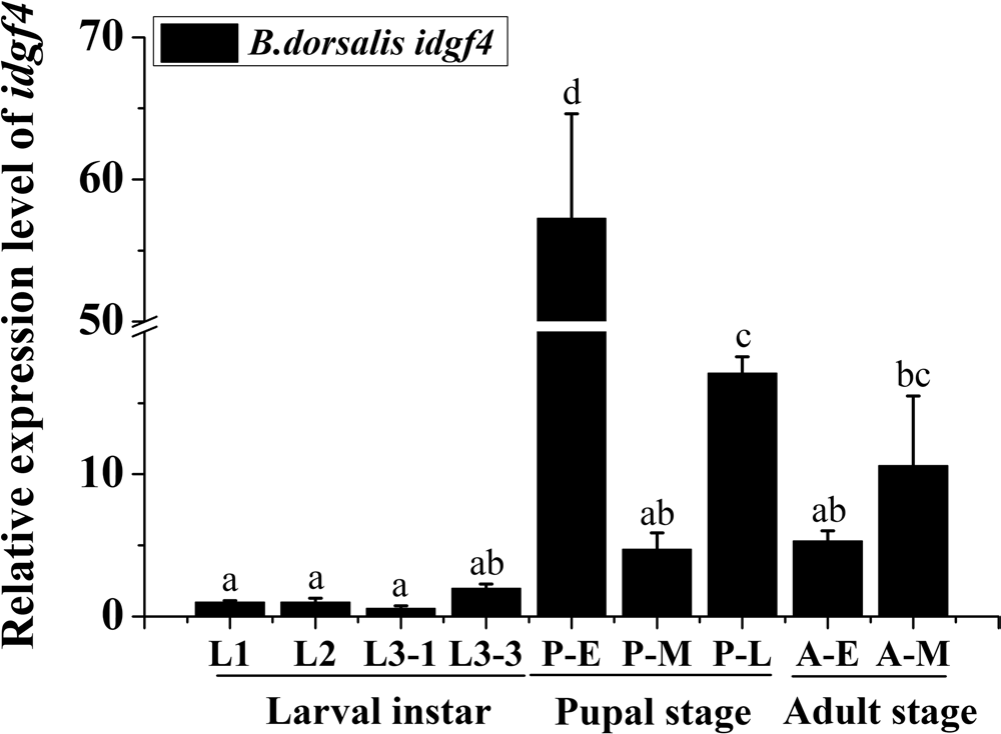
The expression of *idgf4* at nine developmental stages of *B. dorsalis*. The nine developmental stages examined include the 1^st^ instar larvae (L1), 2^nd^ instar larvae (L2), 3^rd^ early-instar larvae (L3-1), 3^rd^ instar larvae (L3-3), 1–3 days mixed pupae as early pupae (P-E), 4–6 days as medium pupae (P-M), and 7–9 days as late pupae (P-L), 1-day adults (A-E) and 10-day adults (A-M). The results are presented as the relative expression after normalization against the endogenous *18s rRNA* gene. Expression is relative to the gene expression in 1^st^ instar larvae (assigned a value of 1). Different letters above the bars represent significant differences at *P*  < 0.01, as determined by Tukey’s HSD tests.

The expression of *idgf4* differed significantly in certain developmental stages (Tukey HSD tests: P < 0.05). In the larval stage, *idgf4* was expressed in the 1^st^ instar and tended to stabilize until the late third instar. In the pupal stage, the highest level of mRNA expression was detected in early pupae (P = 0.000), then declined during medium pupae and recovered to the second highest level of expression in late pupae (P = 0.000). In adults, the relative expression of *idgf4* on day 10 was significantly higher than that on day 1 (P = 0.029) and the third highest expression level was measured in the 10-day-old adults. Also, *idgf4* expression differ significantly among the other stages, i.e., the expression levels of larval stages (L1, L2, L3-1) compared to L3-3, P-M, and A-E (Fig. 3). The different expression levels indicate that *idgf4* has special physiological roles in different developmental stages.

### Silencing of *idgf4* caused the low heat tolerance and morphogenesis in *B. dorsalis*

#### In the functional study of the larval stage

**Figure 4:**
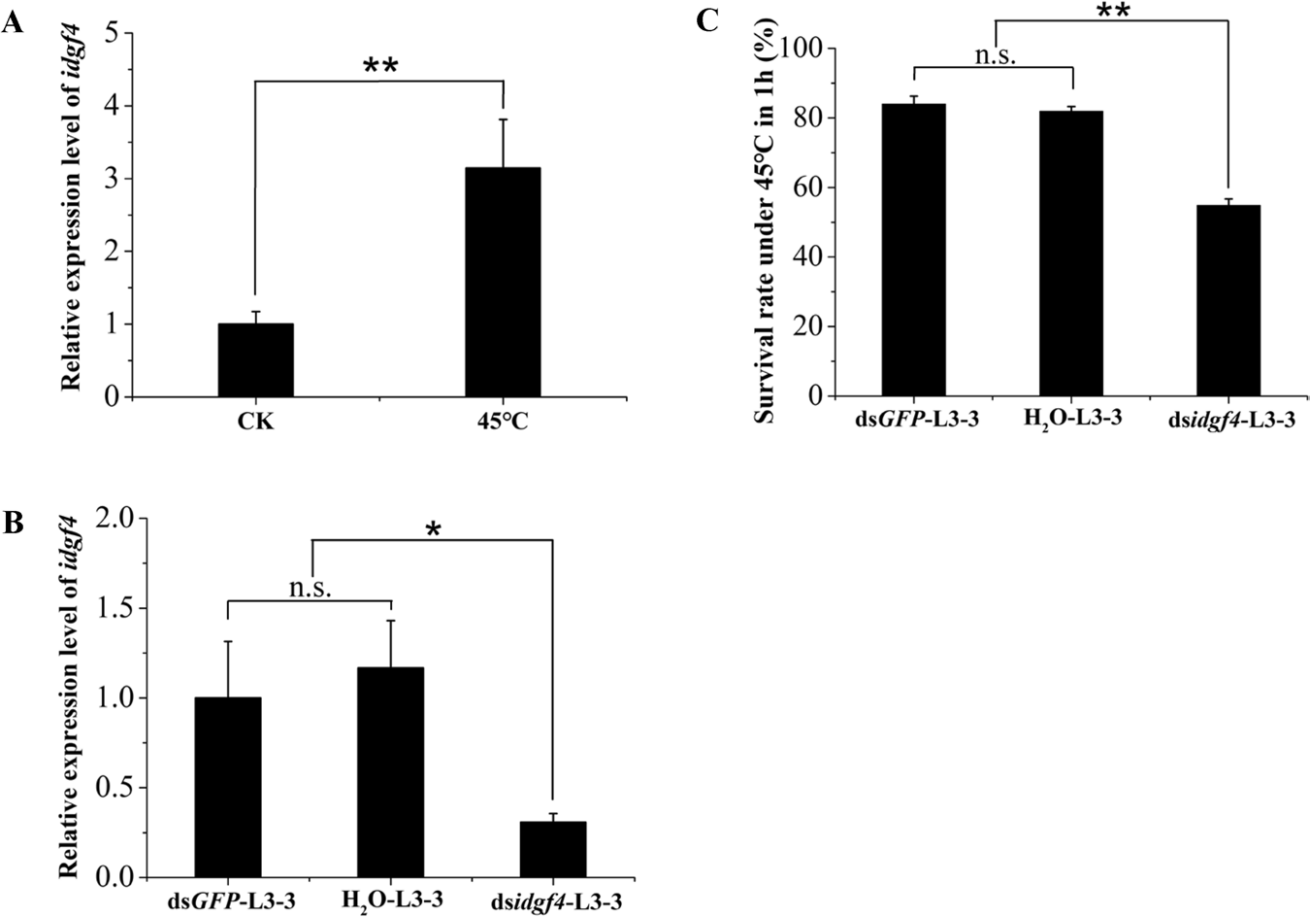
Effect of heat treatment on *B. dorsalis* larvae. (**A**) Relative expression level of *idgf4* in 3^rd^ instar larvae (7-day larvae) at 45 °C for 1 h. (**B**) The relative expression level of *idgf4* after feeding ds*idgf4*. (**C**) The survival rate of *B. dorsalis* under high temperature treatment after silencing. All the fruit flies in the function study in (**B**,**C**) were 5-day-old 3^rd^ early-instar larvae exposed to dsRNA at 1000 ng/µl concentration or H_2_O for 48 h at 25 °C. ** indicates a statistically significant difference in *idgf4* mRNA expression and survival rate between the treatment group and the control ds *GFP* and H_2_O groups (*P*  < 0.01, t-test). * indicates a statistically significant difference in *idgf4* expression between the ds*idgf4* group and the control ds*GFP* and H_2_O groups (*P*  < 0.05, t-test). “n.s.” indicates no statistically significant difference in *idgf4* expression between the control ds*GFP* and H_2_O groups.

First, we tested the expression level of *idgf4* under extremely high temperatures without ds*idgf4* feeding. In the extreme high temperature group (45 °C for 1 hour followed by 4 h at 25 °C), the *idgf4* expression level of insects that survived successfully was 3.15 times higher compared to the control group (25 °C for 5 h) (t-test: P = 0.012, Fig. 4A). We wondered what would happen if we decreased the expression of *idgf4*, so RNAi was used to study the response after a decline in *idgf4* expression. After 5-day-old 3^rd^ early-instar larvae of *B. dorsalis* were exposured to ds *idgf4* at 1000 ng/µl concentration for 48 hours, the target gene *idgf4* showed 69.2% and 73.6% silencing compared to d*sGFP*-feeding and H_2_O-feeding groups, respectively (t-test: P = 0.047 and 0.011, Fig. 4B). Afterwards, the survival rate was detected under the treatments of 45 °C for 1 hour followed by 4 h at 25 °C. Compared to the ds*GFP*-feeding and H_2_O-feeding groups, the survival rates decreased by 29.1% and 27.1%, respectively (Tukey’s HSD test: P = 0.000, Fig. 4C).

#### In the functional study of the pupal stage

**Figure 5:**
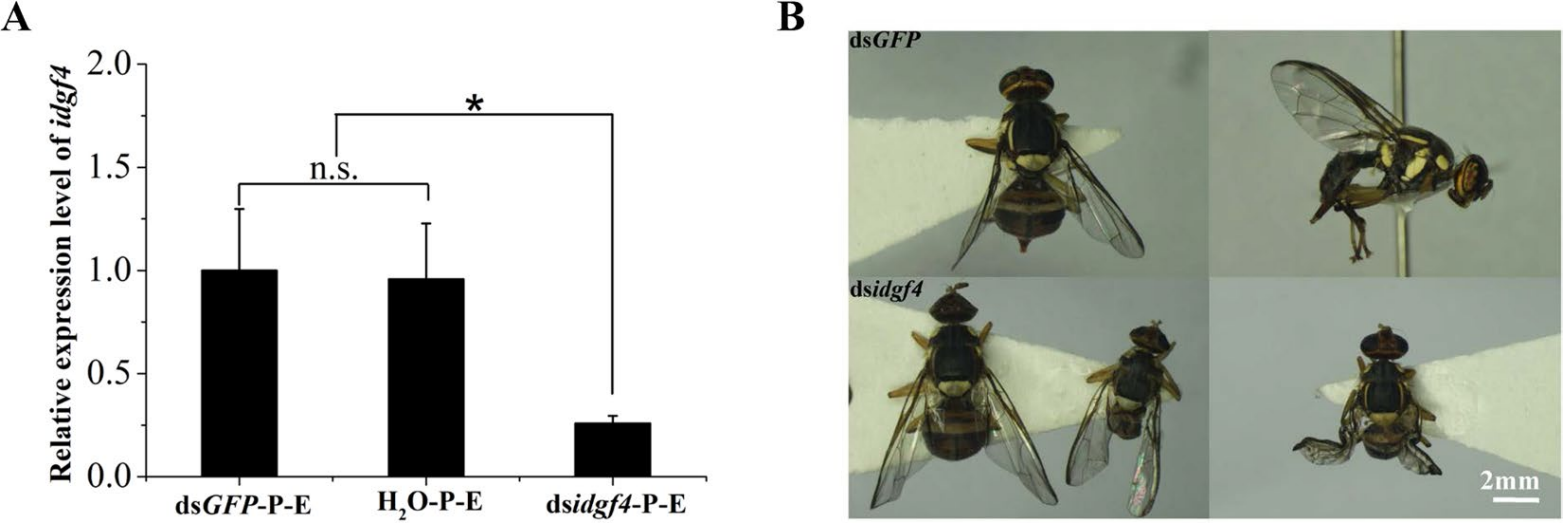
Effect of silencing *idgf4* in *B. dorsalis*. (**A**) The relative expression level of *idgf4* after feeding ds*idgf4* in early pupae. (**B**) Malformed individuals after the silencing. All the fruit flies in the function study in Fig. 5 were 5-day-old 3^rd^ early-instar larvae exposed to dsRNA at 1000 ng/µl concentration or H_2_O for 96 h at 25 °C. * indicates  a statistically significant difference in *idgf4* mRNA expression between the ds*idgf4* group and the control ds*GFP* and H_2_O groups (*P*  < 0.05, t-test). “n.s.” indicates no statistically significant difference in *idgf4* expression between the control ds*GFP* and H_2_O groups.

After 5-day-old 3^rd^ early-instar larvae of *B. dorsalis* were exposed to ds*idgf4* at 1000 ng/µl concentration for 96 h at 25 °C, the target gene *idgf4* showed 74.1% and 73.0% silencing compared with ds*GFP*-feeding and H_2_O-feeding groups, respectively (t-test: P = 0.037 and 0.036, Fig. 5A), through the expression level detection in the early pupal stage using qRT-PCR. We found approximately 17.5% of individuals per treatment were deformed after all the fruit flies emerged, exhibiting two types of malformation. One type caused smaller fruit flies, and the other resulted in partly extensible wings, which led to a loss of flight capacity (Fig. 5B and Table S3). During subsequent development, approximately 100% of deformed adults died before sexual maturity, approximately 5 days after emergence. In addition, there were no dead or malformed flies in the control including the H_2_O and ds*GFP* feeding groups, and all the flies lived for more than half a month.

## Discussion

The poorly described GH 18 family is a key gene family in insect development, with members, such as chitinases, having catalytic activity, as well as some proteins without catalytic activity, such as IDGFs^46^. We have been interested in one gene *idgf4* in this family to study its role in insect defense barrier against heat and other key functions during the development in Tephritid fruit fly species for the first time. There are predicted and conserved domains in the *idgf4* protein containing the GH 18- IDGF domain, glyco-18 domain (glyco-hydro-18 domain) and Chi A domain (Fig. 1). Two types of the genes with three same domains as same as the phylogenetic tree showed that the genes were highly conserved in the Tephritid fruit flies. However, the *idgf4* of two groups are not paralogous, which indicated different phylogenetic relationship between these two types. Besides the overlapping traits, there were no similarity and dissimilarity between two groups. IDGFs have an eight-stranded alpha/beta barrel fold which are concerned with the GH 18 chitinases, but they have a known amino acid substitution to eliminate chitinase catalytic activity^25^. The Chi A domain may indicate it evolved from chitinases and gain new functions as growth factors with the interaction of cell surface glycoproteins^25,27^. Characteristics such as similar arrangement of introns and exons, small size, and different cytological localization make this family an excellent candidate for evolutionary studies^28^. Previous studies, based on the estimated catalytic sites, found *idgf* family polypeptides with mammalian chitinase-related proteins and confirmed these genes to be homeotic^25,47^. The exon/intron structures of *idgf* genes in six forms of *Drosophila* were also compared^28^. However, these studies did not provide any further details about the members of the *idgf* gene family or certain genes among insects. Using *idgf4* nucleotide sequences of *Drosophila* and Tephritidae in GenBank, we applied the neighbor-joining method and maximum-likelihood method to obtain a phylogenetic tree. *Idgf4* in *B. dorsalis* has high identity with the homologues in other Tephritid fruit flies (Figs 2 and S1). The highly conserved trait reveals the importance of encoding glycosyl hydrolases among similar species and analogous roles in ECM dynamics across the insect taxa^22^. *Idgf*s are structurally related to chitinases such as the Chi A domain suggesting that the family of *idgf*s likely evolved from chitinases^25,27^. However, they acquired a new growth-promoting function, interacting with cell surface glycoproteins implicated in growth-promoting processes.

The *idgf4* gene in *B. dorsalis* is continuously expressed from 1^st^ instar larvae through pupae and adults. However, it mainly expresses in the early and late pupal, and mature adult stages. The expression pattern of *idgf4* in different developmental stages indicates that it may play distinct roles during insect growth and development. This observation is similar to the previous study in which *idgf* genes were expressed at rather constant levels throughout larval and pupal development in *Drosophila*^22^. UAS-RNAi-mediated knockdown of the individual *idgf4* gene displayed serious epidermal lesions at wounded sites of 1^st^ instar larvae and the wounded epidermal cuticle barrier lost integrity^22^. Although a large number of studies have paid more attention to its expression in the larval stage, in *B. dorsalis*, it is mainly expressed in the early and late pupal stages as well as adult stages^22,35^. The knockdown studies in *Drosophila* identified *idgf* genes involved in cuticle molting during the pupal stage, and the gene reduction could cause pupal lethality^22^. In general, we chose pupal stages when the *idgf4* gene was expected to play important roles on development as the highest expression levels, to study the function of the *idgf4* gene^22^.

The ECM, whose formation involves *idgf4*, functions as a local defense barrier against a hostile environment; here we studied the gene function of *idgf4* in extreme temperature defense^22^. We used an extremely high temperature to test the gene function in protection of larvae from a hostile environment. The development and growth of insects are greatly affected by environmental temperatures due to their poor ability to adjust and maintain their body temperature as poikilotherms^45^. Among all the stages, larvae were chosen because they have a strong selection for heat resistance and plasticity as they are exposure to thermal stress inside the fruits^45^. The inadequate environment of high temperature leads to an increased expression of *idgf4* in surviving larvae. The increasing expression pattern of this gene might reveal its involvement in heat tolerance. Besides, the comparative transcriptome analyses also indicated *idgf4* may play some roles in the adaptation^45^. We decreased the expression of *idgf4* using RNAi to study its effect on heat acclimation. After the insects were fed with ds*idgf4* for 48 h, the expression level of *idgf4* decreased significantly. Meanwhile, the survival rate after the feeding also had a significant drop. In previous research, the *idgf4* gene was required for exoskeletal barrier function that led to the normal development of a healthy cuticle throughout larval development^22^. The *idgf* genes also showed a potential function in insect temperature adaptation in desert beetle Microdera punctipennis, in which low temperatures, such as 4 °C and −4 °C could upregulate the expression of six genes, including *idgf2*, in the GH 18 family^48^. We showed the same results; that high temperature can upregulate the expression of *idgf4* and that silencing this gene can decrease the survival rate under heat stress in *B. dorsalis*. Our results also indicated that the expression of *idgf4* can help to increase the heat tolerance (Fig. 4). Although the inside of fruits actually provides insects with a much more stable environment than soil, where pupae develop, the larval stage was still chosen to study the heat influence. In our previous study, the 3^rd^ instar larval stage was found to be the most sensitive stage to heat^45,49^. However, compared to the larvae, pupae could withstand a great range of temperature changes based on the survival rate after different temperature treatments in *Bactrocera* species^50^.

Some studies certified that the *idgf4* gene is related to the growth and development of insects^22,25,27,36^. This gene is involved in chitin-based cuticle development, imaginal disc development and wound healing in *D. melanogaster* according to Flybase (www.flybase.org). The histological analysis of third instar larval individuals, in which *idgf4* had been knocked down, showed unusually narrowed ECM thickness and deformed ECMs^22^. In addition, gene function screening via RNAi in *T. castaneum* showed that the *idgf4* gene was required for adult eclosion, but had no effect on adult female fecundity or fertility^31^. In our research, the expression of *idgf4* was highest in the early and late pupal stages, which indicated its possible function for insect growth and development during the pupal stage (Fig. 3). Although all the treated individuals emerged after the silencing, approximately 17.5% of malformed individuals were found in the ds*idgf4*-feeding group, including smaller and abnormally extensible winged adults that could not survive to sexual maturity (Fig. 5). The imperfect development implied that they had weak diffusion and competition ability which led to the loss of flight ability compared to the healthy flies. In addition, in all the functional studies, two control groups, including irrelevant dsRNA of *GFP* and H_2_O without any dsRNA, were used, and no difference were found between these two different control groups. No dead or malformed flies in the control groups indicated that the malformed development was related to the *idgf4* gene knockdown, not the RNAi methodology itself. The miniaturization of their body size and the evolution of flight and wings, which enhance the ability of insects to colonize novel ecological habitats, profoundly influences all aspects of their biology, from development to behavior^51^. In insects, body size affects important fitness variables such as mate selection, predation and tolerance to heat, cold and starvation^52^. In addition, miniaturization imposes steep demands on the flight system because smaller insects must flap their wings at higher frequencies to generate sufficient aerodynamic forces to stay aloft^51^. During pupation in *Drosophila*, the development of imaginal discs and the wing is a key developmental period while the insect is in a compacted form^53^. These results also revealed that this gene in *B. dorsalis* had the same function in the development of wings and also the same expression pattern as the one in silkworms, whose mRNA copies of *idgf* were less during the early pupal stage in scaleless mutants^35^. These changes in wings might make it impossible for fruit flies to disperse and reproduce.

*B. dorsalis* has shown a remarkable range expansion over the past 10 years and has invaded several new continents^5^. With global warming in recent years, *B. dorsalis*, poses a significant risk to both agriculture and the environment. Recently, the dsRNA designed to target pest genes has emerged as a promising strategy for improving pest control^54^. In this study, we demonstrated that the expression of the *idgf4* gene in *B. dorsalis* was significantly decreased by feeding insects the dsRNA of *idgf4*. Considering its function in the larval and pupal stages, *idgf4* can be used as a potential target gene for pest control. Although we reduced the expression of this gene in early pupal period, nearly 20% of *B. dorsalis* were unable to recover, even after 10 days of pupation in our study. The irreparability of wings and body size, as well as the highly conserved traits, indicate that *idgfs* are key genes for pest.

Despite the economic importance of *B. dorsalis*, only limited information regarding the molecular and developmental biology of this insect exists^55^. Here, we report the expression pattern and function of one gene in a poorly described *idgf* family in depth. It is widely accepted that strong heat tolerance and healthy growth contribute to further invasion ability^45^. After the *idgf4* knockdown experiment, the decreased survival ability under high temperature in larvae and malformed individuals in pupae indicated the function of this gene in invasive fruit fly development and heat tolerance. Additionally, our results provide new insights into the function of *idgf* family members and may reveal a new potential gene for pest control.

## Methods

### Insects

Individuals of *B. dorsalis* were collected from their original invaded province of Guangdong and reared as described by Yuan *et al*.^56,57^. All the fruit flies were maintained at 25 °C, with a 14 h light: 10 h dark photoperiod. Eggs and larvae were given an artificial diet consisting of 120 g sugar, 40 g yeast extract, 10 g peptone, 10 g agar, 8.8 g antibiotic and 1000 ml H_2_O. The adult flies were cultured using 25% sucrose and 75% peptone. The fruit flies had been maintained in the laboratory for approximately 10 generations prior to the experiments to eliminate temperature plastic traits derived from local environmental influence. Two hundred individuals were cultured in a 45 cm*45 cm*50 cm insect rearing cage and three cages totally were used to culture the fruit flies. We also put 10–50 new field fruit flies from Guangzhou to each cage every half year.

### RNA extraction and cDNA synthesis

Each stage had five replicates for *idgf4* expression pattern detection and every replicate with different number of individuals (per replication: L1 50 larvae, L2 40 larvae, L3-1 and L3-3 30 larvae, pupal stage including P-E, P-M and P-L 6 pupae, adult stages including A-E and A-M 10 adults) were collected randomly and mixed together. We distinguish each stage based on the body size and the development of oral hooks through microscope according to methods provide by Zhou’s study^58^. The mouth hook length and width for the 1^st^ (0.0846 ± 0.0005 mm, 0.0116 ± 0.0003 mm), 2^nd^ (0.1512 ± 0.0013 mm, 0.0308 ± 0.0007 mm) and 3^rd^ instars (0.2913 ± 0.0015 mm, 0.0677 ± 0.0013 mm) were distinct, respectively. The average body length of the 1^st^, 2^nd^ and 3^rd^ instar larvae were 0.9725 ± 0.0275 mm, 3.2011 ± 0.0961 mm and 7.9726 ± 0.2006 mm. The average body width of the 1^st^, 2^nd^ and 3^rd^ instar larvae were 0.2111 ± 0.0049 mm, 0.5882 ± 0.0181 mm and 1.4113 ± 0.0309 mm. Based on the body size and oral hooks, we can know the exact developing time for each larvae stage under our culturing condition^58^. RNAi efficiency was detected with five replicates in the larval stage and four replicates in the pupal study, and one replicate containing 10 randomly collected larvae or pupae and multiple insects together for sampling. RNA was extracted from the whole body of larvae, pupae and adults using the RNAsimple Total RNA Kit (Tiangen, China). cDNA was synthesized from 1000 ng total RNA using PrimeScript^@^ RT reagent Kit with gDNA Eraser (Perfect Real Time) (Takara, Japan) following the manufacturer’s instructions.

### ORF cloning of *B. dorsalis idgf4*

To verify the ORF of *idgf4* in *B. dorsalis*, three pairs of primers were designed based on the conserved regions of the *idgf4* from *B. oleae*, *C. capitata*, *D. melanogaster* (sequence from GeneBank) and the sequence of *idgf4* from the result of the *B. dorsalis* transcriptome (No. SRP093863). The primers for cloning and amplification conditions are shown in Tables 1 and S2, respectively. The ORF and conserved domain were identified with the ORF Finder software (http://www.ncbi.nlm.nih.gov/gorf/gorf.html) and NCBI BLAST results (http://blast.ncbi.nlm.nih.gov/Blast.cgi). The isoelectric point and molecular weight were predicted using the SWISS-MODEL (https://web.expasy.org/compute_pi/).

**Table 1:** Primers used for cloning and real time qRT-PCR amplification.

Gene	Primer	Sequence	Size (bp)
*Bd 18s rRNA-rt*	*18s-rt-F*	GCGAGAGGTGAAATTCTTGG	160
	*18s-rt-R*	CGGGTAAGCGACTGAGAGAG	
*Bd EFα1-rt*	*EFα1-rt-F*	CGTTGGTGTCAACAAGATGG	230
	*EFα1-rt-R*	TGCCTTCAGCATTACCTTCC	
*Bd GAPDH-rt*	*GAPDH -rt-F*	GACGCCTACAAGCCTGACAT	221
	*GAPDH -rt-R*	GTTGAAGCGGGAATGATGTT	
*Bd RPL13-rt*	*RPL13-rt-F*	CAGTTGTACGTTGCGAGGAAT	134
	*RPL13-rt-R*	TCTTGATGGAGCACGGGAG	
*Bd idgf4-rt*	*idgf4-rt-F*	ATGTTCCTCCGCCTTCT	152
	*idgf4-rt-R*	CGCATACCGTTCATAAATAG	
*Bd cloneidgf4-1*	*idgf4idgf4-whole seq-F-1*	GAAATTTGTCATTTTGCTAGC	1300
	*idgf4-whole seq-R-1*	GGCACGTAAGATGGGATA	
*Bd cloneidgf4-2*	*idgf4-whole seq-F-2*	TTACTTTGGAGGCGAGTAGTTC	341
	*idgf4-whole seq-R-2*	CATTGCTGCTGACAAGTTTATT	
*Bd cloneidgf4-3*	*idgf4-whole seq-F-3*	TTGGGTGGGCTATGACGA	265
	*idgf4-whole seq-R-3*	ATGCTCCGAACAATGAAACT	
*Bd dsidgf4*	*idgf4-dsRNA-F*	CATAGCGGCATCGGTAAAT	574
	*idgf4-dsRNA-R*	GAACCATCAGCGCCTTCA	

### Phylogenetic analysis

The integrity of homologous amino acid sequences of other species was retrieved from the NCBI server. Sequences were first aligned by the conserved sequences and then phylogenetic analysis was performed using two methods: the neighbor-joining method in the Molecular Evolutionary Genetics Analysis software (MEGA version 5.1) and the maximum-likelihood method in the RAxML analysis^59^. ModelGenerator 0.85 was used to find the optimal amino acid substitution model and the LG + G model was selected^60^. One thousand bootstrap iterations were conducted to obtain branch support values. All the positions that contained gaps and missing data were eliminated before alignment and phylogenetic analysis.

### qRT-PCR detection of gene expression

qRT-PCR was performed using SYBR® Premix Ex Taq ™ II (Tli RNaseH Plus) (Takara, Japan) on an ABI 7500 instrument (USA). All RNA samples were analyzed in triplicate (tech reps). The reactions included 1 µl cDNA, 12.5 µl SYBR Green mix, 1 µl each of forward and reverse primers, 0.5 µl ROX Reference Dye II and 9 µl ddH_2_O. The thermocycler conditions were 95 °C for 30 s, followed by 40 cycles at 95 °C for 5 s and 52 °C for 34 s. Melting curve analysis was performed at the end of each expression analysis, using the following conditions: 95 °C for 15 s, followed by 52 °C for 60 s. Four genes including *18s rRNA*, *RPL13*, *EFα1* and *GAPDH* were tested the stability to be used as endogenous reference genes by geNorm and *18s rRNA* was selected (Fig. S2)^45,61,62^. The sequences of the qRT-PCR primers used for the reference and target genes are described in Table 1.

### Expression pattern of *idgf4*

To determine the constitutive expression of *idgf4*, fruit flies were collected at the following nine specific developmental stages: 2-day-old 1^st^ instar larvae, 4-day-old 2^nd^ instar larvae, 5-day-old 3^rd^ early-instar larvae, 7-day-old 3^rd^ instar larvae, 1–3 days mixed pupae (early pupae), 4–6 days (medium pupae), 7–9 days (late pupae), 1-day adults (early adults), and 10-day adults (late adults). Every replicate with different number of individuals (see RNA extraction and cDNA synthesis) were collected randomly and mixed together for *idgf4* expression pattern detecting. Meanwhile, the 5-day-old 3^rd^-early instar larvae of *B. dorsalis* were collected for the dsRNA feeding experiment. After RNA extraction and cDNA synthesis, the expression pattern was detected using qRT-PCR. Each stage had five replicates.

### dsRNA preparation for gene function study

Double-stranded RNA of *idgf4* (ds*idgf4*) was used to knock down *idgf4* expression, and double-stranded RNA of green fluorescent protein (ds*GFP*) and H_2_O without any dsRNA were used as the negative controls. The dsRNAs were synthesized by using the T7 RiboMAX Express RNAi system (Promega, USA). The primers for dsRNA synthesis and amplification conditions are shown in Tables 1 and S2, respectively. The 5-day-old 3^rd^-early instar larvae of *B. dorsalis* were collected and moved into a 50 ml tube with 3 holes on the lid. In the functional study of the larval stage, five replicates were performed for each treatment and each replicate contained 40 larvae. Three grams of artificial diet material with 30 μl of a dsRNA solution or H_2_O was used for feeding for 48 h. In the functional study of the pupal stage, four replications were performed for each treatment and each replication contained 30 larvae. Three grams of artificial diet material with 30 μl of a dsRNA solution or H_2_O was used for feeding for 96 h. The concentration of the dsRNA solution for the primary exposure was 1000 ng/μl.

### Heat treatments

To study the effect of *idgf4* expression on the temperature tolerance of insects, 30 individuals (7-day-old 3^rd^ instar larvae after hatching) were selected for the temperature treatments without dsRNA feeding. Larvae were exposed to 45 °C, which was selected as the thermal stress endpoint, for 1 h and experienced mortality in the range of 40–50%^45^. After exposure to the heat stress, the larvae were returned to 25 °C, and their survival rate was scored after 4 h to prevent insects from heat-shock fainting. A larva was considered dead if no movement was detected when being poked gently by forceps. The survived insects were immediately killed using liquid nitrogen, and stored at -80 °C for further molecular analysis. Then, for the H_2_O and dsRNA-feeding larval temperature study, forty larvae (5-day-old 3^rd^ early-instar larvae) were fed an artificial diet supplemented with H_2_O and dsRNA for 48 h. Ten larvae were randomly selected and immersed in the liquid nitrogen to kill for RNA isolation. The remaining 30 individuals (7-day-old 3^rd^ instar larvae: after feeding the ds*GFP*, H_2_O and ds*idgf4* for 48 h, all 5-day-old 3^rd^ early-instar larvae come 7-day-old 3^rd^ instar larvae) were chosen for the temperature treatments as mentioned before. All larvae were exposed to 45 °C for 1 h. After exposure to the heat stress, the larvae were returned to 25 °C and their survival rate was scored after 4 h. The larva was considered dead using the method mentioned before. During the thermal treatments, samples were enclosed in 2 ml tubes with a hole on the lid, were topped with 4 g diet and incubated in a water bath set to the desired temperature using a PolyScience Programmable Temperature Controller (USA). Each treatment (ds*GFP*, H_2_O and ds*idgf4*) included five biological replicates.

### Silencing *idgf4* in *B. dorsalis*

For the adult malformation study, thirty 5-day-old 3^rd^ early-instar larvae were fed with ds*GFP*, H_2_O and ds*idgf4* for 48 h and transferred to a new artificial diet with the same treatment for another 48 h. After 96 h, the larvae developed to maturity and were then transferred into soil for pupation. After one to three days of pupation, ten early pupae were killed for the RNAi efficiency detection. The remaining twenty individuals were continued to be fed as adults and were used for phenotype observation on the 2^nd^ day after emergence. The mortality of emerged individuals was recorded in twenty days after the flies emerged. Each treatment (ds*GFP*, H_2_O and ds*idgf4*) included four biological replicates.

### Statistical analysis

The qRT-PCR data were analyzed using the 2−ΔCT method^63^. The expression of *idgf4* was quantified in the larvae treated with ds*GFP*, H_2_O and ds*idgf4*. Biological replicates were used for statistical analysis. All results from experimental replicates were analyzed using Student’s t-test or a Turkey’s test using SPSS 20 (IBM Corporation, USA).

## Supplementary information

Supplementary information

## Data Availability

All data generated or analyzed during this study are included in this published article (and its supplementary information file).
