# Supplementary material for: Imaginal disc growth factor 4 regulates development and temperature adaptation in Bactrocera dorsalis
Source: Sci Rep. 2019 Jan 30;9:931. doi: 10.1038/s41598-018-37414-9 (PMC6353879; doi:10.1038/s41598-018-37414-9)
Supplement: Supplementary file 1 — Supplementary information [file 41598_2018_37414_MOESM1_ESM.pdf]

Imaginal disc growth factor 4 regulates development and temperature adaptation in *Bactrocera dorsalis*

Xinyue Gu, Zhihong Li, Yun Su, Yan Zhao and Lijun Liu*

[1]Department of Entomology, College of Plant Protection, China Agricultural University, Beijing 100193, China.

*: Correspondence and requests for materials should be addressed to Lijun Liu. (email: ljliu@cau.edu.cn)

Supplementary information

Figure S1. Phylogenetic analysis of *idgf4* from different insect species using Neighbor-Joining and Maximum-Likelihood methods**.**

Figure S2. Stability of four housekeeping genes of *B. dorsalis* in the whole bodies of larvae at different developmental stages evaluated by *geNorm*.

Table S1. Details of *IDGF4* protein sequences used for phylogenetic analysis.

Table S2. PCR amplification conditions.

Table S3. Details of silencing *idgf4* in the function study of pupal stage.

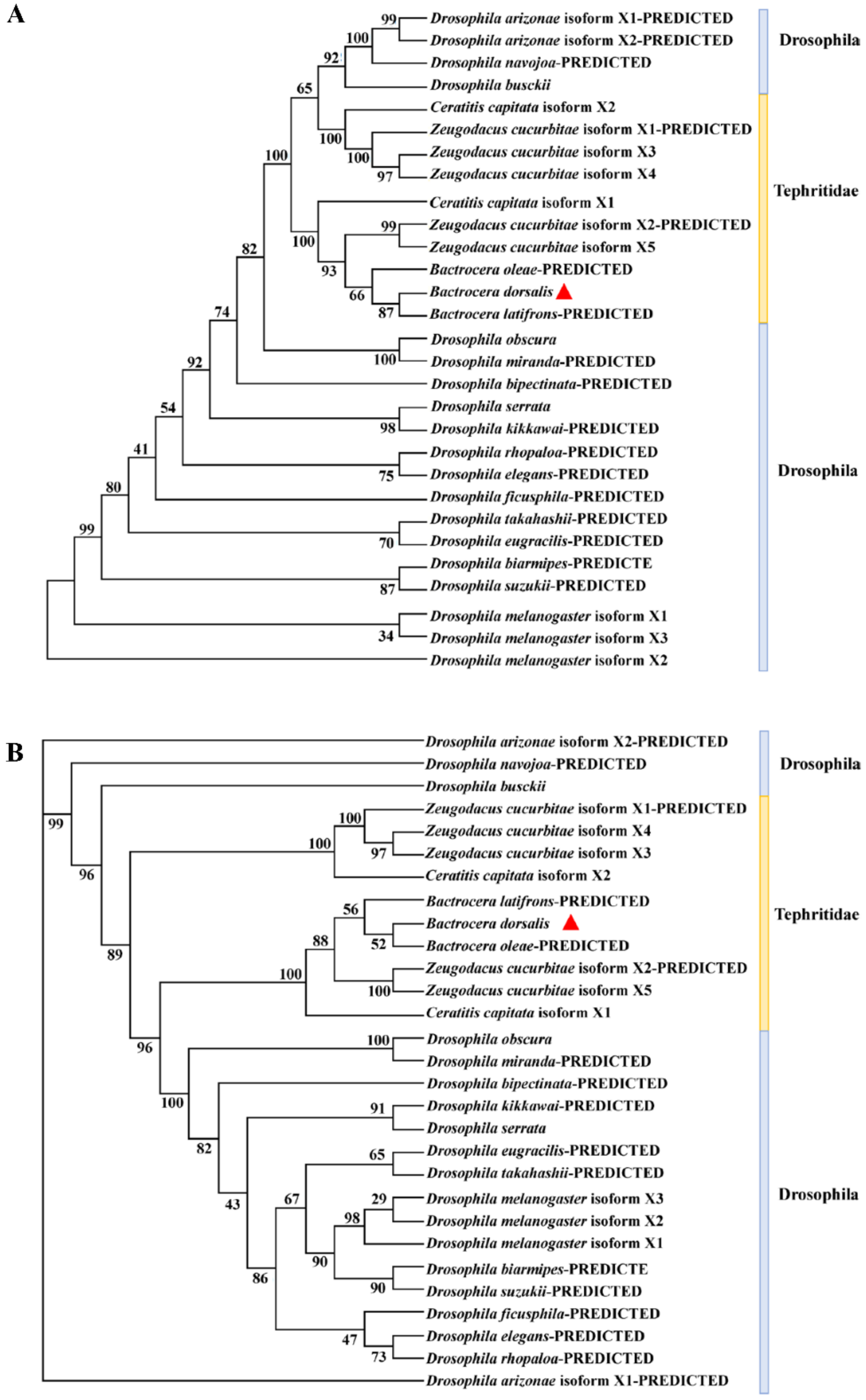

Figure S1. Phylogenetic analysis of *idgf4* from different insect species using Neighbor-

Joining and Maximum-Likelihood methods**. A.** The phylogenetic analysis of *idgf4* using the Neighbor-Joining method in MEGA. B. The phylogenetic analysis of *idgf4* using Maximum-Likelihood method in RAxML. One thousand bootstrap replications were used to test the topology in all cases. Percentage bootstrap values were reported on each cluster. The *B. dorsalis idgf4* we got is labeled with a red triangle. The amino acid and nucleotide sequences were downloaded from NCBI. The accession numbers of the genes are designated with the corresponding abbreviations and are listed in Table S1.

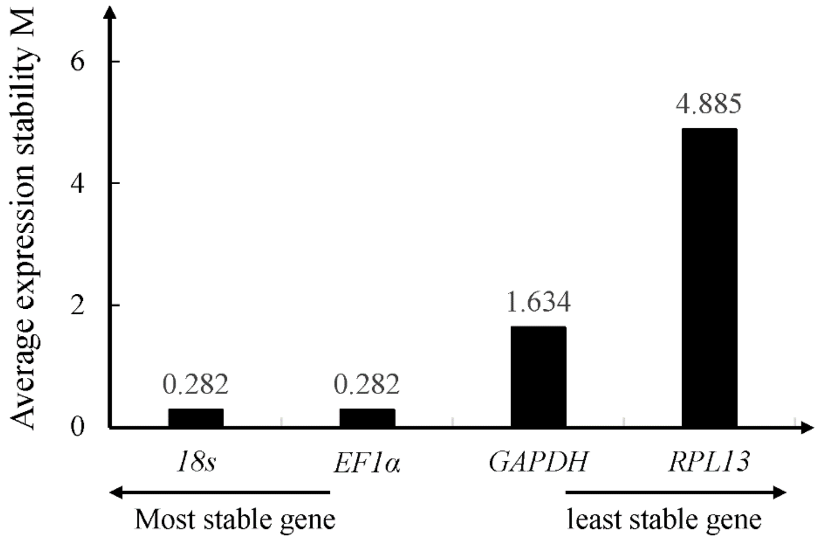

Figure S2. Stability of four housekeeping genes of *B. dorsalis* in the whole bodies of larvae at different developmental stages evaluated by *geNorm*. Four different developmental stages were used including 1st-instar larvae, early 3rd instar larvae (L3-1), 3-day pupae (P-E) and 10-day adults (A-M).

Table S1. Details of *IDGF4* protein sequences used for phylogenetic analysis.

No.	Species	Name of *idgf4*	Accession Number
1	*Bactrocera dorsalis*	*Bactrocera dorsalis*	MH250169
2	*Bactrocera latifrons*	*Bactrocera latifrons*-PREDICTED	XP_018800655.1/<br>JAI32325.1
3	*Bactrocera oleae*	*Bactrocera oleae*-PREDICTED	XP_014099295.1
4	*Ceratitis capitata*	*Ceratitis capitata* isoform X1	JAB95810.1/<br>XP_004521633.1
5	*Ceratitis capitata*	*Ceratitis capitata* isoform X2	XP_004520935.3
6	*Drosophila arizonae*	*Drosophila arizonae* isoform X1-PREDICTED	XP_017872388.1
7	*Drosophila arizonae*	*Drosophila arizonae* isoform X2-PREDICTED	XP_017872390.1
8	*Drosophila biarmipes*	*Drosophila biarmipes*-PREDICTE	XP_016954605.1
9	*Drosophila bipectinata*	*Drosophila bipectinata*-PREDICTED	XP_017094827.1
10	*Drosophila busckii*	*Drosophila busckii*	ALC48324.1
11	*Drosophila elegans*	*Drosophila elegans*-PREDICTED	XP_017114436.1/<br>XP_017114435.1
12	*Drosophila eugracilis*	*Drosophila eugracilis*-PREDICTED	XP_017064046.1
13	*Drosophila ficusphila*	*Drosophila ficusphila*-PREDICTED	XP_017055733.1
14	*Drosophila kikkawai*	*Drosophila kikkawai*-PREDICTED	XP_017030292.1
15	*Drosophila melanogaster*	*Drosophila melanogaster* isoform X1	AAF46534.1
16	*Drosophila melanogaster*	*Drosophila melanogaster* isoform X2	AAN09618.1
17	*Drosophila melanogaster*	*Drosophila melanogaster* isoform X3	AHN59538.1
18	*Drosophila miranda*	*Drosophila miranda*-PREDICTED	XP_017156091.1
19	*Drosophila navojoa*	*Drosophila navojoa*-PREDICTED	XP_017965606.1
20	*Drosophila obscura*	*Drosophila obscura*	XP_022234159.1
21	*Drosophila rhopaloa*	*Drosophila rhopaloa*-PREDICTED	XP_016988593.1
22	*Drosophila serrata*	*Drosophila serrata*	XP_020802057.1
23	*Drosophila suzukii*	*Drosophila suzukii*-PREDICTED	XP_016937309.1
24	*Drosophila takahashii*	*Drosophila takahashii*-PREDICTED	XP_017012977.1
25	*Zeugodacus cucurbitae*	*Zeugodacus cucurbitae* isoform X1-PREDICTED	XP_011189060.1
26	*Zeugodacus cucurbitae*	*Zeugodacus cucurbitae* isoform X2-PREDICTED	XP_011185355.1
27	*Zeugodacus cucurbitae*	*Zeugodacus cucurbitae* isoform X3	JAD07329.1
28	*Zeugodacus cucurbitae*	*Zeugodacus cucurbitae* isoform X4	JAD02610.1
29	*Zeugodacus cucurbitae*	*Zeugodacus cucurbitae* isoform X5	JAD00079.1

Table S2. PCR amplification conditions.

**Primers pair**	**Amplification conditions**
18s rRNA- rt &<br>*Bd EFα1-rt &*<br>*Bd RPL13-rt&*<br>*Bd GAPDH-rt*<br>*idgf4-rt*	95 ℃ for 30 s; 40 cycles at 95 ℃ for 5 s, 52 ℃ for 34 s; 95 ℃ for 15 s; 52 ℃ for 60 s
Bd cloneidgf4-1	95 ℃ for 2 min; 40 cycles at 95 ℃ for 30 s, 52 ℃ for 30 s, 72 ℃ for 60 s; 72 ℃ for 10 min
Bd cloneidgf4-2	95 ℃ for 2 min; 40 cycles at 95 ℃ for 30 s, 56 ℃ for 30 s, 72 ℃ for 60 s; 72 ℃ for 10 min
Bd cloneidgf4-3	95 ℃ for 2 min; 40 cycles at 95 ℃ for 30 s, 56 ℃ for 30 s, 72 ℃ for 60 s; 72 ℃ for 10 min
Bd dsidgf4	95 ℃ for 2 min; 40 cycles at 95 ℃ for 30 s, 54 ℃ for 30 s, 72 ℃ for 60 s; 72 ℃ for 10 min

Table S3. Detail number of individuals on silencing *idgf4* in the function study of pupal stage per replicate.

treatment	total number of insects per replicate	successful pupation per replicate	average of deformed individuals per replicate	average of smaller individuals per replicate	average of deformed wings per replicate	died individuals before sexual maturity
idgf4	20	20	3.50±0.96	1.50±0.50	2.00±0.82	3.50±0.96
GFP	20	20	0	0	0	0
H_2O	20	20	0	0	0	0
